# Application of a Tool to Identify Undiagnosed Hypertension — United States, 2016

**DOI:** 10.15585/mmwr.mm6729a2

**Published:** 2018-07-27

**Authors:** Elizabeth L. Ciemins, Matthew D. Ritchey, Vaishali V. Joshi, Fleetwood Loustalot, Judy Hannan, John K. Cuddeback

**Affiliations:** ^1^American Medical Group Association (AMGA), Alexandria, Virginia; ^2^Division for Heart Disease and Stroke Prevention, CDC.

Approximately 11 million U.S. adults with a usual source of health care have undiagnosed hypertension, placing them at increased risk for cardiovascular events (*1*–*3*). Using data from the National Health and Nutrition Examination Survey (NHANES), CDC developed the Million Hearts Hypertension Prevalence Estimator Tool, which allows health care delivery organizations (organizations) to predict their patient population’s hypertension prevalence based on demographic and comorbidity characteristics (*2*). Organizations can use this tool to compare predicted prevalence with their observed prevalence to identify potential underdiagnosed hypertension. This study applied the tool using medical billing data alone and in combination with clinical data collected among 8.92 million patients from 25 organizations participating in American Medical Group Association (AMGA) national learning collaborative* to calculate and compare predicted and observed adult hypertension prevalence. Using billing data alone revealed that up to one in eight cases of hypertension might be undiagnosed. However, estimates varied when clinical data were included to identify comorbidities used to predict hypertension prevalence or describe observed hypertension prevalence. These findings demonstrate the tool’s potential use in improving identification of hypertension and the likely importance of using both billing and clinical data to establish hypertension and comorbidity prevalence estimates and to support clinical quality improvement efforts.

This study used medical billing^†^ and electronic health record (EHR) clinical data collected among 8.92 million patients, aged 18–85 years, who had ≥1 ambulatory office visit for evaluation and management in 2016 within one of 25 AMGA-member organizations. These organizations use the Optum One population health analytic tool^§^ and pool billing and clinical data as part of a national learning collaborative.

Observed hypertension prevalence was defined using three case definitions that use increasing amounts of billing and clinical data collected during the observation year. The first hypertension case definition included patients with at least one diagnosis code for hypertension^¶^ on a billing claim. Patients without a diagnosis code on a billing claim, but who had a diagnosis code for “hypertension” on their EHR problem list** met the hypertension criteria for the second case definition. Additional patients were added who did not meet criteria for the first two case definitions, but who had elevated in-office blood pressure (BP) readings, defined as a single reading ≥160/100 mm Hg or two readings on different days ≥140/90 mm Hg. The first and second case definitions reflect documented diagnoses of hypertension. The BP criteria in the third case definition align with national recommendations for diagnosing hypertension^††^; however, patients who meet this definition alone are not considered to have a hypertension diagnosis and might not have hypertension upon further assessment.

Predicted hypertension prevalence was determined by applying the Hypertension Prevalence Estimator Tool to the organizations’ data; development and validation of the tool are described elsewhere (*2*). The tool requires input of the patient population’s demographic characteristics (i.e., distribution by sex, race/ethnicity, and age group) with the option^§§^ of providing the prevalence of three comorbidities within the patient population that aid in predicting hypertension prevalence (i.e., the presence of none, one, or two or more of the following conditions: obesity, diabetes, and chronic kidney disease). Similar to identifying hypertension, comorbidities were identified during the observation year using 1) medical billing claims only^¶¶^; 2) problem list diagnosis codes; or 3) other clinical data.***

The observed hypertension prevalence and the 95% confidence intervals of the predicted hypertension prevalence, calculated with and without use of organization-specific information on comorbidity prevalence, were compared overall and by organization using each case definition.

A total of 8.92 million patient records were included, with patient populations ranging from 50,000 to 1.02 million across the 25 organizations. Nearly 40% of patients were aged 45–64 years; 57% were female, and 74% were non-Hispanic white (range = 47%–90%) (Table 1). Overall, 5.9% of patients with ≥1 office visit during 2016 had no BP reading recorded (range = 0.3%–15.9%) (Supplementary Figure, https://stacks.cdc.gov/view/cdc/54153).

**TABLE 1:** Patient characteristics of 25 health care delivery organizations participating in application of Million Hearts Hypertension Prevalence Estimator Tool — United States, 2016

Characteristic	Overall population	Range
No. of patients included in analyses,* millions	8.92	0.05–1.02
**Age group (yrs), %**		
18–44	34.2	25.6–39.4
45–64	39.5	36.1–42.6
65–74	16.9	13.8–22.9
75–85	9.4	7.4–14.7
**Sex, %**		
Women	57.3	52.6–61.1
Men	42.7	38.9–47.4
**Race/Ethnicity %**		
White, non-Hispanic	73.9	46.9–90.3
Black, non-Hispanic	7.1	0.4–20.2
Hispanic	3.4	0.7–9.4
Other	10.5	1.7–34.9
Missing	5.1	0.4–15.0

*Aged 18–85 years with a least one ambulatory care visit during 2016.

Comorbidity prevalence and predicted and observed hypertension prevalence varied overall and by organization depending on the evidence used (Table 2) (Table 3) (Supplementary Figure, https://stacks.cdc.gov/view/cdc/54153). Overall obesity prevalence increased from 10.7% using billing data alone to 45.0% using all three data sources (Table 2). Use of billing data alone indicated that 4.4% of patients had 2–3 comorbidities; the addition of problem list data alone and in combination with other clinical data increased detection of 2–3 comorbidities to 5.7% and 14.3%, respectively. Prevalence of 2–3 comorbidities ranged from 8.3% to 18.1% across organizations using all three data sources.

**TABLE 2:** Variation in observed and predicted hypertension prevalence with increasing levels of medical billing and clinical data used, overall and across health care delivery organizations (HDOs) (n = 8.92 million) participating in application of Million Hearts Hypertension Prevalence Estimator Tool — United States, 2016

Prevalence	Overall total			Range across HDOs*		
	Claims	Claims or problem list	Claims with problem list and clinical criteria	Claims	Claims or problem list	Claims with problem list and clinical criteria
**Comorbidity prevalence, %**						
Obesity	10.7	13.1	45.0	4.6 to 34.7	7.2 to 35.2	29.6 to 51.4
Diabetes	11.3	12.9	16.4	6.0 to 13.8	6.8 to 17.5	9.2 to 21.8
Chronic kidney disease	3.4	4.4	7.4	1.2 to 5.2	1.4 to 6.3	3.6 to 9.3
Combined prevalence of the above conditions						
0 conditions	79.4	76.2	48.3	59.6 to 86.5	58.2 to 84.4	41.5 to 63.7
1 condition	16.3	18.1	37.5	11.2 to 31.5	12.8 to 32.5	27.4 to 42.4
2–3 conditions	4.4	5.7	14.3	2.3 to 8.9	2.8 to 9.3	8.3 to 18.1
**Hypertension prevalence**						
Observed, %	29.1	30.0	36.0	17.1 to 35.4	18.3 to 37.8	24.2 to 46.1
No. (millions)	2.60	2.68	3.21	0.02 to 0.05	0.02 to 0.06	0.03 to 0.07
Predicted^†^ using organization-specific comorbidity data, % (95% CI)	33.2 (33.2–33.3)	33.9 (33.9–34.0)	39.5 (39.5–39.5)	30.2 to 40.1	30.9 to 41.4	35.5 to 47.6
Percentage point difference,^§^ (95% CI)	4.1 (4.1–4.2)	3.9 (3.9–4.0)	3.5 (3.5–3.6)	0.0 to 14.7	0.4 to 13.9	1.0 to 13.8
No. of additional patients identified	366,000	348,000	312,000	24 to 65,000	731 to 67,100	267 to 57,700
Predicted^†^ not using organization-specific comorbidity data,^¶^ % (95% CI)	38.5 (38.5–38.6)	38.5 (38.5–38.6)	38.5 (38.5–38.6)	35.4 to 46.2	35.4 to 46.2	35.4 to 46.2
Percentage point difference,^§^ (95% CI)	9.4 (9.4–9.5)	8.5 (8.5–8.6)	2.5 (2.5–2.6)	-21.1 to 4.0	-19.9 to 2.8	-14.0 to 2.8
No. of additional patients identified	838,000	758,000	223,000	2,910 to 119,000	1,770 to 114,000	130 to 57,800

**Abbreviation**: CI = confidence interval.

* Range of values calculated across the 25 health care delivery organizations participating in the American Medical Group Association's national learning collaborative; 95% CIs are not provided for the predicted hypertension prevalence estimates.

^†^ Based on Million Hearts Hypertension Prevalence Estimator Tool.

^§^ Compared with observed prevalence. Observed prevalence was always less than predicted prevalence.

^¶^ The comorbidity profile of the health care delivery organization’s patient population is estimated using National Health and Nutrition Examination Survey databased on the organization’s patient population’s age, gender, and race/ethnicity characteristics.

**TABLE 3:** Observed and predicted prevalence of hypertension among the American Medical Group Association's member health care delivery organizations — United States, 2016

Organization	Medical claims only*		Medical claims plus problem list*		Medical claims plus problem list plus clinical data*		Based on national comorbidity estimates^†^
	Observed^§^	Predicted^¶^	Observed^§^	Predicted^¶^	Observed^§^	Predicted^¶^	Predicted^¶^
1	35.4%	40.1%	37.8%	41.4%	46.1%	47.6%	46.2%
2	34.9%	38.5%	35.5%	38.9%	44.3%	44.6%	43.9%
3	34.6%	39.0%	37.0%	39.3%	40.4%	42.4%	40.7%
4	34.2%	34.2%	35.4%	35.0%	41.0%	40.0%	38.2%
5	31.9%	32.4%	32.3%	33.3%	39.3%	40.4%	37.9%
6	31.8%	33.6%	32.6%	34.3%	40.7%	40.1%	38.0%
7	31.4%	34.2%	31.4%	35.0%	38.5%	41.1%	40.8%
8	30.5%	31.5%	30.7%	32.2%	34.9%	36.8%	36.1%
9	30.1%	35.9%	31.5%	36.7%	37.5%	42.2%	40.6%
10	29.6%	35.0%	30.9%	35.3%	38.5%	39.8%	39.1%
11	28.9%	31.1%	29.9%	31.7%	36.8%	38.6%	36.3%
12	28.6%	32.5%	29.2%	33.3%	33.8%	38.4%	37.9%
13	28.5%	32.6%	29.8%	33.5%	34.7%	39.3%	38.1%
14	28.4%	32.3%	29.9%	32.9%	39.3%	40.0%	38.4%
15	28.4%	34.0%	32.9%	34.9%	37.3%	40.8%	39.5%
16	28.3%	30.9%	29.7%	31.7%	33.6%	37.1%	35.4%
17	28.3%	35.4%	28.8%	36.2%	35.0%	41.3%	41.3%
28	28.0%	35.3%	28.9%	35.9%	33.2%	40.0%	41.4%
19	27.5%	30.2%	27.7%	30.9%	33.8%	37.0%	35.9%
20	27.5%	32.9%	28.6%	33.7%	33.5%	39.3%	38.0%
21	24.7%	34.0%	27.2%	34.4%	35.7%	40.7%	39.9%
22	24.5%	32.4%	25.7%	32.7%	30.7%	37.1%	37.5%
23	24.2%	33.1%	24.3%	33.7%	31.4%	39.3%	38.4%
24	22.2%	31.4%	22.7%	31.8%	26.5%	35.5%	37.8%
25	17.1%	31.8%	18.3%	32.2%	24.2%	38.0%	38.2%

* Observed prevalence of the three comorbidities within the organizations’ patient population is used to predict hypertension prevalence. Comorbidities were identified based on: 1) “medical claims only”: at least one diagnosis code for the condition on an outbound billing claim (*International Classification of Disease, Tenth Revision*, *Clinical Modification* [ICD-10-CM] code of E66.09, E66.1, E66.8, E66.9, E66.01, E66.2, Z68.3X, Z68.4X, Z68.54, or R93.9 for obesity; E10.X or E11.X for diabetes; and I12.X, I13.X, or N18.X for chronic kidney disease); 2) “medical claims plus problem list”: adds additional patients who had a diagnosis code for obesity, diabetes, or chronic kidney disease on their electronic health record (EHR) problem list (same codes as designated for claims); and 3) “medical claims plus problem list & clinical data”: adds additional patients who had a body mass index ≥30 kg/m^2^ for obesity; hemoglobin A1c of ≥6.5%, plasma glucose of ≥126 mg/dL, fasting plasma glucose of ≥126 mg/dL, or a glucose tolerance test of ≥200 mg/dL for diabetes; and an estimated glomerular filtration rate of <60 mL/min per 1.73 m^2^ for chronic kidney disease.

^†^ Predicted prevalence of the three comorbidities within the organizations’ patient population is used to predict hypertension prevalence. Predicted comorbidity prevalence is estimated based on the organization population prevalence of age, gender, and race/ethnicity characteristics and use of National Health and Nutrition Examination Survey data. Using this method does not affect the observed hypertension prevalence; therefore, no observed prevalence values are provided.

^§^ Defined using: 1) “medical claims only”: at least one diagnosis code for hypertension on an outbound billing claim ( ICD-10-CM code of I10, I11.X, I12.X, or I13.X); 2) “medical claims plus problem list”: adds additional patients who had a diagnosis code for “hypertension” on their EHR problem list (same codes as designated for claims); and 3) “medical claims plus problem list & clinical data”: adds additional patients who had elevated in-office blood pressure readings, defined as a single reading ≥160/100 mm Hg or two readings on different days ≥140/90 mm Hg.

**^¶^** Determined by applying the Million Hearts Hypertension Prevalence Estimator Tool to the organizations’ data. The predicted hypertension prevalence is estimated based on the distribution of patients by age, gender, race/ethnicity, and predicted or diagnosed comorbidity prevalence (presence of 0, 1, or 2–3 of the following conditions: obesity, diabetes and chronic kidney disease).

With the addition of each data source to identify hypertension and the comorbidities, overall observed hypertension prevalence increased from 29.1% to 30.0% to 36.0% (range = 2.60–3.21 million patients), and overall predicted hypertension prevalence increased from 33.2% to 33.9% to 39.5% (range = 2.96–3.52 million patients), respectively (Table 2) (Table 3) (Supplementary Figure, https://stacks.cdc.gov/view/cdc/54153). Differences between the estimates for observed and predicted hypertension prevalence ranged from 3.5 to 4.1 percentage points, representing a range of 312,000 to 366,000, or one in eight to one in 11 patients who potentially have undiagnosed hypertension. Across the 25 organizations, observed hypertension prevalence ranged from 24.2% to 46.1%, predicted hypertension prevalence ranged from 35.5% to 47.6%, and the difference between the two ranged from 1.0 to 13.8 percentage points, with predicted prevalence always higher than observed prevalence.

Removing organization-specific comorbidity data from the information used to predict hypertension prevalence and relying on the NHANES-based comorbidity estimates provided in the Estimator Tool resulted in an overall predicted hypertension prevalence of 38.5% and increased the difference between observed and predicted prevalence from 2.5 to 9.4 percentage points, depending on the data sources used to identify hypertension (Table 2).

## Discussion

Application of the Million Hearts Hypertension Prevalence Estimator Tool using billing and clinical data collected from approximately 9 million U.S. adult patients within multispecialty medical groups and integrated systems across the country revealed that up to one in eight patients with hypertension might not have received a diagnosis. Across the 25 organizations assessed, the difference between predicted and observed hypertension prevalence was as high as 13.8 percentage points, and the percentage of patients with an outpatient visit who did not have a documented BP measurement during the observation period was as high as 15.9%. The identification of lower than anticipated hypertension prevalence or BP screening rates allows organizations to evaluate and refine systems of care to improve the diagnosis and management of hypertension (*3*). This could include, as an initial step, reassessing patients who had a single in-office BP ≥160/100 mm Hg or two readings on different days ≥140/90 mm Hg to establish, if warranted, a documented diagnosis and to ensure provision of appropriate hypertension treatment. This is a conservative approach, and recent guidelines (*4*) might suggest even lower thresholds. One report found that approximately one in three patients who met the BP criteria alone and were able to be reassessed received a diagnosis of hypertension (*5*).

This report reinforces the utility of using multiple data sources to identify patients in potential need of chronic disease management and to estimate the prevalence of chronic conditions. In addition, these findings indicate how the identification of patients for inclusion in clinical registries or quality improvement measure reporting^†††^ depend on the data types (i.e., medical billing data alone or in combination with clinical data) used to detect the targeted conditions. Higher comorbidity and observed hypertension prevalence were found when clinical data were included with billing data for case ascertainment, particularly for obesity. Billing data are generated to initiate payment for services rendered, and some conditions might not be prioritized for treatment or billing because of patients’ competing health needs or limited reimbursement. Therefore, use of billing data alone to describe the prevalence of hypertension and other chronic conditions or to predict hypertension prevalence likely underrepresents the burden (*6*–*8*). If organizations are unable to use all three data sources to describe their comorbidity prevalence (in particular obesity prevalence), they might consider using the nonorganization-specific comorbidity estimates provided in the Hypertension Prevalence Estimator Tool to predict their hypertension prevalence. When the nonorganization specific comorbidity estimates were applied, the predicted hypertension prevalence typically was closest to the observed hypertension prevalence determined using all available billing and clinical data.

The findings in this report are subject to at least four limitations. First, the billing and clinical definitions used align with national standards and guidelines, but variation might exist in how the conditions are diagnosed and documented across organizations. Furthermore, the data were not assessed to ensure appropriate coding or documentation. Both of these factors could potentially lead to variation in disease prevalence estimates, including the degree of prevalence underestimation, and indicate more differences in clinical practice, documentation, and billing than in the actual health status of the population. Second, organizations participating in this national learning collaborative are considered to be high performing; therefore, the differences between predicted and observed hypertension prevalence reported in this study are likely to underestimate quality gaps in other organizations. Third, it was not possible to determine the actual observed hypertension prevalence of this population. To do so would involve further assessment of those patients who either met the clinical definition alone or did not have a BP assessment during the observation period. Finally, new evidence suggests that compared with standardized BP observation, BP readings taken in a clinical setting overestimate systolic BP by an average of 6.4 to 11.8 mm Hg depending on the study setting and independent of “white coat syndrome” or masked hypertension (*9*,*10*)

Improving management of hypertension in health care organizations is multifaceted, requiring interventions across multiple systems and within diverse disciplines, including those reviewed in the Guide to Community Preventive Services^§§§^ and summarized by the Million Hearts initiative.^¶¶¶^ The tool assessed in this report can be used to support the evaluation of the effectiveness of these organizations in identifying hypertension. With recently released updated hypertension guidelines (*4*) that increased the number of persons classified as having hypertension, there is an urgent need for careful and thorough identification and treatment of people with hypertension.

**Summary**

**What is already known about this topic?**

Approximately 11 million U.S. adults with a usual health care source have undiagnosed hypertension. Identification, diagnosis, and treatment of hypertension are needed to decrease the risk for an adverse cardiovascular event.

**What is added by this report?**

Using the Million Hearts Hypertension Prevalence Estimator Tool to calculate and compare observed and predicted prevalences of hypertension among approximately 9 million U.S. patients revealed that nearly one in eight patients with hypertension might not have received a diagnosis.

**What are the implications for public health practice?**

The Hypertension Prevalence Estimator Tool might improve hypertension identification within health care delivery organizations; using both billing and clinical data to establish hypertension and comorbidity prevalence estimates are important to support clinical quality improvement efforts.

## Footnotes

* All AMGA Analytics for Improvement (A4i) participants use the Optum One population health and risk analytics platform. Optum collects longitudinal data on approximately 95 million patients and 175 million claims. These data represent a subset of this larger database. The 25 organizations included in this analysis are AMGA members that vary in size (70–3,000 FTE physicians) and serve in total approximately 25 million patients; 52% are integrated delivery systems. Among the 8.92 million adult patients aged 18–85 years assessed in this study, 51% had commercial health insurance, 36% had some form of Medicare coverage, 5% had Medicaid coverage, 0.9% were uninsured, and 7% had some other form of health insurance or were missing information (<1%). Additional information about the learning collaborative is available at http://www.amga.org/wcm/A4I/wcm/AboutAMGA/CF/Anceta/A4I/index_a4i.aspx?hkey=9429bbc5-72d9-4eb7-9cfa-f80d8bc40cc2.

† Billing data consist of outbound administrative claims, collected longitudinally.

§ https://www.optum.com/solutions/prod-nav/performance-analytics.html.

¶ *International Classification of Diseases* (ICD), *Tenth Revision*, *Clinical Modification* (ICD-10-CM) code of I10, I11.X, I12.X, or I13.X (reflects ICD, *Ninth Revision,* Clinical Modification (ICD-9-CM) codes of 401.X, 402.X, 403.X, or 404.X).

** Same codes as designated for claims to identify hypertension or the three comorbidities. Organizations typically use ICD-10-CM codes in their problem list documentation, including all 25 AMGA member organizations. Some organizations might use other nomenclature (e.g. SNOMED-CT) or still rely on text-based fields.

†† The blood pressure thresholds used align with those recommended in the Seventh Report of the Joint National Committee on Prevention, Detection, Evaluation, and Treatment of High Blood Pressure (JNC-7) (https://www.ncbi.nlm.nih.gov/books/NBK9630/), and the ≥140/90 mm Hg threshold to identify hypertension is used by Million Hearts for national hypertension surveillance and in the development of the Hypertension Prevalence Estimator Tool.

§§ Organizations that do not have access to accurate comorbidity data are still able to use the Million Hearts Hypertension Prevalence Estimator Tool (https://nccd.cdc.gov/MillionHearts/Estimator/). If no accurate comorbidity data are available, the comorbidity profile of the organization’s patient population is estimated using NHANES data that are based on the organization’s patient population’s age, gender, and race/ethnicity characteristics. If limited accurate comorbidity data are available, organizations can use their own data for some conditions (e.g., diabetes and chronic kidney disease), but allow the tool to provide estimates for the other conditions (e.g., obesity) using NHANES data that are based on their patient population’s age, gender, and race/ethnicity characteristics.

¶¶ ICD-10-CM codes of E66.09, E66.1, E66.8, E66.9, E66.01, E66.2, Z68.3X, Z68.4X, Z68.54, or R93.9 for obesity (reflects ICD-9-CM codes of 278.00, 278.01, 278.03, V85.3X, V85.4X, V85.54, or 793.91); ICD-10-CM codes of E10.X or E11.X for diabetes (ICD-9-CM: 250.X); and ICD-10-CM codes of I12.X, I13.X, or N18.X for chronic kidney disease (ICD-9-CM: 403.X, 404.X, or 585.X).

*** Body mass index ≥30 kg/m^2^ for obesity; hemoglobin A1c of ≥6.5%, fasting plasma glucose of ≥126 mg/dL, plasma glucose conducted on the same day as a lipid panel of ≥126 mg/dL (assumes fasting), or a glucose tolerance test of ≥200 mg/dL for diabetes; and an estimated glomerular filtration rate of <60 mL/min per 1.73 m^2^ for chronic kidney disease.

††† For example, inclusion in hypertension registries or in the National Quality Forum’s Controlling High Blood Pressure Measure (NQF 0018).

§§§ https://www.thecommunityguide.org/content/task-force-findings-cardiovascular-disease.

¶¶¶ https://millionhearts.hhs.gov/tools-protocols/hiding-plain-sight/toolkit.html.
